# Young women were associated with higher risk of hypertensive disorders of pregnancy and cesarean section from hormone replaced cycles in frozen-thawed embryo transfer: a retrospective study of 5316 singleton deliveries

**DOI:** 10.3389/fendo.2023.1238887

**Published:** 2023-09-05

**Authors:** Xinyao Hu, Zhiqi Liao, Jie Li, Yueping Zhou, Yaxin Guo, Kun Qian

**Affiliations:** Reproductive Medicine Center, Tongji Hospital, Tongji Medical College, Huazhong University of Science and Technology, Wuhan, China

**Keywords:** frozen-thawed embryo transfer, birthweight, endometrial preparation, perinatal outcome, hormone replacement treatment (HRT)

## Abstract

**Background:**

The utilization of frozen-thawed embryo transfer (FET) cycles has been linked to heightened risks of adverse perinatal outcomes. However, the potential association between adverse perinatal outcomes and distinct endometrial preparation regimens remains unclear. Therefore, we aim to investigate the maternal and neonatal outcomes after hormone replacement treatment (HRT) cycles, natural cycles (NC) and HRT cycles with pretreatment using GnRHa (HRT + GnRHa) for ovulatory women undergoing FET cycles.

**Methods:**

A large sample retrospective cohort study was carried out from 2016 to 2020. The data included a total of 5316 women who had singleton deliveries undergoing FET cycles and which were divided into three groups based on different endometrial preparation protocols: 4399 patients in HRT groups, 621 in GnRHa+HRT groups, 296 in NC groups. The outcomes consisted of maternal outcomes (cesarean section, hypertensive disorders of pregnancy (HDP), placenta previa, gestational diabetes mellitus (GDM));and neonatal outcomes (preterm birth, newborn birthweight, low birthweight, small for gestational age (SGA), macrosomia, large for gestational age (LGA), fetal malformation).

**Results:**

After adjusting for a series of confounding variables, we found an increased risk of HDP (aOR=3.362; 95%CI, 1.059-10.675) and cesarean section (aOR=1.838; 95%CI, 1.333-2.535) in HRT cycles compared with NC, especially for ovulatory women under 35 years old. However, in all three groups, newborn birth weight was not significantly different. Meanwhile, perinatal outcomes did not differ significantly in terms of perinatal outcomes in HRT +GnRHa cycles compared with HRT cycles solely.

**Conclusion:**

During FET cycles, singletons from HRT were related to higher risks of HDP and cesarean section, particularly for young women. GnRHa pretreatment didn’t bring any benefit to perinatal outcomes compared with HRT cycles alone. Therefore, the natural cycle may be a more appropriate and safer option for young ovulatory women.

## Introduction

Since a live birth following frozen-thawed embryo transfer (FET) was reported in 1984 firstly, FET has been increasingly applied in assisted reproductive field around the world (1, 2). Initially, this approach was designed for those ovarian hyper-responders to mitigate the hazards of ovarian hyperstimulation syndrome (OHSS), while also providing patients time for fertility preservation and preimplantation genetic testing (PGT) (3, 4). Previous studies made comparations between fresh embryo transfer (ET) and FET, with findings indicating a noteworthy rise in live birth rates (LBR) for FET, especially for high responders. However, FET carries an elevated risk of pre-eclampsia and large for gestational age (LGA) when considering the safety of FET (3, 5, 6). The underlying factors remain unclear and are worth noting.

Recent studies have examined the variance in perinatal outcomes among patients utilizing different endometrial preparation methods during FET cycles (7–10). These findings drew conflicting conclusions of the optimal endometrial preparation approach. In general, hormone replacement treatment (HRT) cycles and natural cycles (NC) are the most frequently employed endometrial regimens for women capable of ovulation during FET. In NC, ovulation occurs naturally, followed by the formation of corpus luteum (CL). In clinical practice, HRT cycles were commonly applied due to their convenience for patients and reduced monitoring requirements for embryo transfers (11). Additionally, HRT cycles can be conducted with or without downregulation induced by gonadotropin-releasing hormone agonist (GnRHa). It has been reported that pituitary suppression through GnRHa administration has a positive impact on patients with adenomyosis (12). However, few randomized controlled trials (RCTs) demonstrated that HRT pretreatment with GnRHa in FET didn’t bring benefit to women with better pregnancy outcomes (13, 14). Consequently, no clear answer has been found in the effect of GnRHa during FET cycles. As of yet, there exists no definitive answer regarding the impact of GnRHa on FET cycles. Furthermore, only a limited number of studies have investigated the influence of various endometrial preparation protocols on the outcomes of singleton neonates, with small sample sizes being a common limitation. What’s more, which endometrial preparation protocols in FET will bring superior perinatal outcomes for ovulatory women remains unclear.

Therefore, the objective of our study was to explore the effect of three different FET endometrial preparation protocols on maternal and neonatal outcomes. To achieve this, a large sample retrospective cohort study was conducted to compare maternal and neonatal outcomes of singleton deliveries after NC, HRT with or without GnRHa for ovulatory women in FET cycles.

## Materials and methods

### Study design and patients

The present study was a retrospective study carried out at the Reproductive Medical Center of Tongji Hospital. The study population comprised patients who underwent FET cycles utilizing three primary endometrial preparation protocols, namely NC, HRT cycles, and GnRHa+HRT cycles, and who delivered live-birth singletons from 2016 to 2020. The inclusion criteria were as follows: body mass index (BMI) < 28 kg/m^2^; maternal age ≤ 42 years; patients whose menstrual cycle is regular (defined as a cycle length between 21 days to 35 days); patients with a singleton birth. Our exclusion criteria were as follows: reduction of multifetal pregnancy; twin delivery with a stillbirth; vanishing twin syndrome (15); patients with congenital uterine malformations; polycystic ovary syndrome (PCOS); patients with chronic hypertension or diabetes mellitus prior to the current pregnancy; intrauterine adhesion (IUA); patients receiving oocytes donation or sperms donation. The present study adhered to rigorous data analysis procedures, we also excluded missing core data from the data analysis, such as unknown gestational week or unclear birthweight. The study obtained the approval of the institutional Review Board of Tongji Hospital, Tongji Medical College, Huazhong University of Science and Technology (TJ-IRB20230394).

### Endometrial preparation before embryo transfer

In our reproductive medicine center, NC, HRT with or without GnRHa cycles were applied to prepare endometrium in FET for ovulatory women according to patients’ condition and physicians’ preferences. In NC group, we measured thickness of endometrium by transvaginal ultrasound and serum progesterone to track follicular development on days 10-12 of menstrual cycle. The ovulation day was determined through analysis of serum luteinizing hormone (LH) level and progesterone level, as well as ultrasound monitoring. When the diameter of the dominant follicle reached 16 mm and serum LH > 20 IU/L with P < 1.0 ng/ml, daily transvaginal ultrasound was performed until ovulation occurred. If the LH level was < 20 IU/L, human chorionic gonadotropin (hCG) was used to trigger ovulation. Subsequently, we thawed and transferred the cleavage embryo three days after ovulation, while blastocyst embryo transfer was executed on the fifth day following ovulation. For luteal phase support, oral dydrogesterone (20 mg, Duphaston; Solvay Phar- maceuticals BV) was used one day after ovulation during natural cycles, a dosage of 40 mg/d dydrogesterone and 200 mg/d progesterone capsules (Utrogestan, Capsugel) were used when endometrial thickness reached 8 mm during HRT cycles. Luteal phase support sustained until 10 weeks of gestation. In HRT group, oral estradiol valerate (Progynova; Bayer Schering Pharma AG, Germany) was given 2 mg/day from cycles days 1 to 4, 4 mg/day from cycles days 5 to 8, 6 mg/day from cycles days 9 to 12. Likewise, we carried out ultrasound examination to measure patients’ ovulation and endometrial thickness from day 13. We adjusted the dose of estradiol dynamically according to patients’ endometrial development status. Once endometrial thickness was ≥ 8 mm, administration of 40 mg progesterone in the way of intramuscular injection was started and continued the next 3 days. FET was then conducted with the guidance of ultrasound on the 4th day of intramuscular progesterone for cleavage embryo, and on the 6th day for blastocyst. In GnRHa+HRT group, the cycles began with 3.75 mg of GnRHa including triptorelin and leuprorelin on day 2 of menstruation. After 28 days of down-regulation, patients conducted the HRT cycles which have been described above.

The overall procedure of embryo culture, vitrification as well as warming has been described previously (16).

### Outcome measures

The present study incorporated outcome data pertaining to gestational weeks, delivery modes, newborn gender, birthweight, obstetric complications, and neonatal diseases, which were retrieved from an electronic medical record database through follow-up procedures.

A positive pregnancy test was confirmed by serum human chorionic gonadotropin (hCG) at two weeks after ET. Clinical pregnancy was defined as the presence of one or more gestational sacs with fetal heartbeat under the guidance of ultrasound. The definition of live birth was the delivery of at least one live newborn child after 24 gestational weeks.

The primary outcomes were maternal outcomes including cesarean section, hypertensive disorder of pregnancy (HDP), placenta previa, gestational diabetes mellitus (GDM). The definition of HDP was on the basis of the International Society for the Study of Hypertension in Pregnancy (ISSHP) (17), including preeclampsia and gestational hypertension. The diagnosis of GDM was according to the consensus described previously (18).

Other outcomes were neonatal outcomes including preterm birth (PTB), defined as gestational age < 37 weeks; very Preterm birth (very PTB), defined as live birth before 32 weeks of gestation (gestational age was calculated from the embryo transfer day to delivery day plus 17 days for cleavage-stage embryo transfer and 19 days for blastocyst transfer); fetal malformation; low birth wight (LBW), defined as birthweight < 2500 g; very low birth wight (very LBW), defined as birthweight < 1500 g; small for gestational age (SGA), defined as birthweight < 10th percentile; macrosomia, defined as birthweight > 4000 g; large for gestational age (LGA), defined as birthweight > 90th percentile; very SGA, defined as birthweight < 3rd percentile; very LGA, defined as birthweight > 97th percentile. SGA, very SGA, LGA and very LGA were defined according to the birthweight reference for Chinese population at different weeks of gestation (19).

### Statistical analysis

In this study, all data analysis were undertaken by SPSS software version 26.0 (SPSS lnc.,Chicago, USA). Shapiro-Wilk test was utilized to measure the distribution of all continuous variables. We presented continuous variables in the form of Mean ± SD or median with interquartile range. Categorical variables were described in the form of frequency and percentage. The analysis of variance (ANOVA) or Kruskal-Wallis test were conducted to assess continuous variables when appropriate. Analysis of categorical variables were performed by Pearson’s chi-squared test or Fisher’s exact test. Bonferroni correction was applied to conduct multiple comparisons.

Using multivariate logistic regressions, we assessed the effect of three endometrium preparation regimens on perinatal outcomes. Covariates that may have an impact on the perinatal outcomes were included which comprised of maternal age at oocyte retrieval, body mass index (BMI), cause of infertility, infertility duration, ART procedure types, the level of FSH, number of transferred embryos, stage of embryo development, PGT treatment. We calculated unadjusted and adjusted odds ratios (ORs) with 95% confidence intervals (CI) to describe the overall association, P values were reported as well. It was considered statistically significant if the P value was less than 0.05.

## Results

### Baseline characteristics

During our study, a total of 5316 patients who met the inclusion and exclusion standards were enrolled. Among these, there were 4399 singletons in HRT group, 621 in HRT + GnRHa group, 296 in NC group. As shown in **Table 1**, the baseline characteristics of the patients were described. For patients’ characteristics, maternal age at oocyte retrieval, basal FSH, AFC, AMH, cause of infertility were significantly different (P<0.05) in three groups. For cycle characteristics, there were statistically significant differences in ovarian stimulation protocols, the number of oocytes retrieval and Metaphase II (MII) oocytes, the number of two pronuclei (2 PN) zygotes, dose and duration of gonadotropin, oocyte maturation rate, the rate of normal fertilization, endometrial thickness, the number and type of embryo transferred in these groups (P<0.05).

**Table 1:** The general characteristics of women with singleton deliveries.

	HRT (n=4399)	GnRH (a)+HRT (n=621)	NC (n=296)	P value
Maternal age at oocyte retrieval, year	30.65 ± 3.95	31.48 ± 3.96	31.14 ± 3.90	<0.001^*^
Body mass index, kg/m^2^	21.31 ± 2.45	21.10 ± 2.41	21.49 ± 2.51	0.056
Basal FSH, mIU/ml	7.29 (6.26,8.50)	7.54 (6.50,8.73)	7.38 (6.45,8.58)	0.004^*^
Antral follicle count	13.0 (9.0,18.0)	10.0 (7.0,15.0)	12.0 (8.0,16.0)	<0.001^*^
AMH, ng/ml	4.26(2.60, 6.87)	3.46(2.04, 5.99)	3.62(2.07, 5.44)	<0.001^*^
Duration of infertility, years	3.0(1.5, 4.0)	3.0 (1.0,4.0)	3.0 (2.0,4.0)	0.468
Type of infertility				0.997
Primary infertility, n (%)	2675 (60.8%)	378 (60.9%)	179 (60.5%)	
Secondary infertility, n (%)	1724 (39.2%)	243 (39.1%)	117 (39.5%)	
Cause of infertility, n (%)				0.005^*^
Female	3015 (68.5%)	433 (69.7%)	195 (65.9%)	
Male	575 (13.1%)	56 (9.0%)	38 (12.8%)	
Mixes	722 (16.4%)	110 (17.7)	60 (20.3%)	
Unexplained	87 (2.0%)	22 (3.5%)	3 (1.0%)	
Ovarian stimulation protocols, n (%)				<0.001^*^
Long GnRHa	1601 (36.4%)	170 (27.4%)	74 (25.0%)	
GnRHa ultra long	1254 (28.5%)	116 (18.7%)	105 (35.5%)	
GnRH antagonist	998 (22.7%)	229 (36.9%)	80 (27.0%)	
Other protocols	546 (12.4%)	106 (17.1%)	37 (12.5%)	
Dose of Gonadotropin, IU	2250.00 (1762.50,2850.00)	2400.00 (2025.00,3000.00)	2366.25 (1875.00,2842.50)	<0.001^*^
Duration of stimulation, days	10.0 (9.0,11.0)	10.0 (9.0,11.0)	10.0 (9.0,11.0)	0.002^*^
The number of oocytes retrieved	14.0 (9.0,19.0)	12.0 (8.0,17.0)	13.0 (9.0,17.0)	<0.001^*^
The number of MII oocytes	12.0 (9.0,17.0)	10.0 (7.0,15.0)	11.5 (8.25,15.0)	<0.001^*^
Oocyte maturation rate (%)	90.91 (81.25,100.00)	88.89 (78.57,100)	91.67 (84.05,100.00)	0.036^*^
ART method, n (%)				0.444
IVF	2789 (63.4%)	400 (64.4%)	192 (64.9%)	
ICSI	1403 (31.9%)	194(31.2%)	97(32.8%)	
Rescue ICSI	207(4.79%)	27 (4.3%)	7(2.9%)	
The number of 2PN	9.0 (6.0,12.0)	8.0 (5.0,11.0)	8.0 (6.0,12.0)	<0.001^*^
PGT treatment, n (%)	134(78.4%)	28(16.4%)	9(5.3%)	0.154
Normal fertilization rate (%)	75.0 (62.5,84.62)	77.778 (65.16,87.29)	72.11 (60.00,83.33)	0.005^*^
Blastocyst formation rate (%)	72.72 (53.33,87.50)	74.17 (57.14,88.22)	7386 (50.00,85.71)	0.180
The number of embryos transferred, n (%)				<0.001^*^
1	3069 (69.8%)	476 (76.7%)	195 (65.9%)	
2	1330 (30.2%)	145(23.3%)	101 (34.1%)	
Stage of embryo development, n (%)				0.002^*^
Cleavage embryo	578 (13.1%)	110(17.7%)	52(17.5%)	
Blastocyst	3821 (86.9%)	511(82.3%)	245 (82.5%)	
Endometrial thickness, mm	9.1(8.4, 10.0)	9.7(8.8,11.0)	9.3(8.4, 10.4)	<0.001^*^

^*^P<0.05.

FSH, follicle stimulating hormone; AMH, Anti Mullerian hormone; GnRH, gonadotropin releasing hormone; ART, assisted reproductive treatment; IVF, in vivo fertilization; ICSI, intracytoplasmic sperm injection; 2PN, two pronuclei; PGT, preimplantation genetic testing.

### Perinatal outcome of singletons

The perinatal outcomes of ovulatory women with singletons who underwent three different endometrial protocols were described in **Table 2**. No significant differences were observed in patients’ gestational week, birthweight, PTB and very PTB among the three groups. The rate of cesarean delivery in NC group (80.1%) was significantly lower than that in HRT + GnRHa group (87.4%) or HRT group (86.9%). The incidence of HDP in HRT group (4.4%) was the highest, while NC group was 1.7% and GnRHa+HRT group was 2.6%. However, the differences were not statistically significant regarding the percentage of GDM, fetal malformation and placenta previa between these groups. Moreover, the incidences of adverse neonatal birthweight including LBW, macrosomia, very LBW, SGA, LGA, very SGA and very LGA demonstrated no significant difference in three groups.

**Table 2:** Maternal and neonatal outcome of singletons based on endometrial preparation regimens.

	HRT	NC	GnRH (a)+HRT	P value
Gestational age, week	39.0 (38.0,39.0)	39.0 (38.0,39.0)	38.5 (38.0,39.0)	0.180
Birthweight, g	3365 (3100,3650)	3370 (3100,3600)	3350 (3100,3650)	0.762
Mode of delivery, n (%)				0.003^*^
Vaginal	57 (13.1)	59 (19.9)	78(10.9)	
Cesarean section	3822(86.9)	237(80.1)	543(87.4)	
Newborn gender, n (%)				0.666
Male	2425(55.1)	159(53.7)	352(56.7)	
Female	1974(44.9)	137(46.3)	269(43.3)	
Preterm birth <37weeks, n (%)	360(8.2)	20(6.8)	56(9.0)	0.506
Very preterm birth <32weeks, n (%)	140(3.2)	5(1.7)	19(3.1)	0.355
Hypertensive disorders of pregnancy, n (%)	193 (4.4)	5 (1.7)	16 (2.6)	0.012^*^
Gestational diabetes mellitus, n (%)	250 (5.7)	21 (7.1)	28 (4.5)	0.271
Placenta previa, n (%)	144 (3.3)	12 (4.1)	21 (3.4)	0.758
Fetal malformation, n (%)	40 (0.9)	2 (0.7)	8 (1.3)	0.590
Low birthweight <2500g, n (%)	193(4.4)	8(2.7)	25(4.0)	0.364
Very low birthweight<1500g, n (%)	22(0.5)	1(0.3)	1(0.2)	0.529
Macrosomia, n (%)	278(6.3)	16(5.4)	37(6.0)	0.784
Small for gestational age, n (%)	194(4.4)	15(5.1)	26(4.2)	0.834
Very small for gestational age, n (%)	68(1.5)	4 (1.4)	9 (1.4)	1.000
Large for gestational age, n (%)	773 (17.6)	44 (14.9)	113 (18.2)	0.445
Very large for gestational age, n (%)	281 (6.4)	14 (4.7)	36 (5.8)	0.461

^*^P<0.05.

Then, we conducted univariate logistic regression analysis of different endometrial protocols in **Table 3**. Furthermore, to compare the association between perinatal outcomes and endometrial preparation protocols, multivariable logistic regression analysis were performed (**Table 4**). After adjusting cofounding variables, we found that both HRT + GnRHa group (aOR=1.697; 95%CI, 1.153-2.497) and HRT group (aOR=1.734; 95%CI, 1.280-2.350) had a significantly increased risk of cesarean delivery in comparison with NC group. Moreover, singletons in HRT group had a higher risk of HDP than that in NC group (aOR=2.980; 95%CI, 1.210-7.335). No significant differences were detected between GnRHa+HRT group and HRT group.

**Table 3:** Univariate logistic regression analysis of perinatal outcomes in different endometrial preparation regimens.

	NC vs HRT		NC vs GnRHa+HRT		HRT vs GnRHa+HRT	
	Crude OR (95%CI)	P value	Crude OR (95%CI)	P value	Crude OR (95%CI)	P value
PTB	1.230(0.771-1.961)	0.384	1.368 (0.805-2.325)	0.247	1.112 (0.828-1.494)	0.481
Very PTB	1.913(0.778-4.705)	0.158	1.837 (0.679-4.968)	0.231	0.960 (0.590-1.562)	0.870
Cesarean section	1.649(1.224-2.222)	0.001^*^	1.733 (1.196-2.511)	0.004^*^	1.051 (0.816-1.353)	0.700
Gender	0.945(0.746-1.196)	0.637	0.887 (0.672-1.171)	0.398	0.939 (0.792-1.112)	0.465
LBW	1.652(0.806-3.384)	0.170	1.510 (0.673-3.389)	0.318	0.914 (0.597-1.399)	0.679
Very LBW	1.483(0.199-11.038)	0.701	0.476 (0.030-7.633)	0.600	0.321 (0.043-2.385)	0.267
Macrosomia	1.181(0.703-1.983)	0.530	1.109 (0.606-2.027)	0.737	0.939 (0.659-1.338)	0.728
SGA	0.864(0.504-1.482)	0.596	0.819 (0.427-1.570)	0.547	0.947 (0.623-1.439)	0.799
Very SGA	1.146(0.415-3.164)	0.792	1.074 (0.328-3.515)	0.907	0.937 (0.465-1.887)	0.855
LGA	1.221(0.878-1.697)	0.235	1.274 (0.872-1.862)	0.211	1.043 (0.839-1.298)	0.702
Very LGA	1.374(0.793-2.383)	0.257	1.240 (0.658-2.336)	0.506	0.902 (0.631-1.290)	0.571
Placenta previa	0.801(0.439-1.461)	0.469	0.828 (0.402-1.707)	0.610	1.034 (0.649-1.648)	0.887
Fetal malformation	1.349 (0.324-5.609)	0.681	1.918 (0.405-9.090)	0.412	1.422 (0.663-3.053)	0.366
HDP	2.671 (1.090-6.541)	0.032*	1.539 (0.558-4.242)	0.404	0.576 (0.344-0.966)	0.037^*^
GDM	0.789 (0.497-1.252)	0.315	0.618 (0.345-1.108)	0.106	0.784(0.525-1.169)	0.232

PTB, preterm birth; LBW, low birth weight; SGA, small for gestational age; LGA, large for gestational age; HDP, hypertensive disorders of pregnancy; GDM, gestational diabetes mellitus; OR, odds ratio.

^*^P<0.05.

**Table 4:** Adjusted ORs for perinatal outcomes in different endometrial preparation regimens by multivariate regression analysis.

	NC vs HRT		NC vs GnRHa+HRT		HRT vs GnRHa+HRT	
	Adjusted OR (95%CI) ^1^	P value	Adjusted OR (95%CI) ^1^	P value	Adjusted OR (95%CI) ^1^	P value
PTB	1.275 (0.797-2.038)	0.311	1.422 (0.823-2.457)	0.208	1.094(0.812-1.474)	0.555
Very PTB	1.999 (0.811-4.925)	0.132	2.076 (0.743-5.801)	0.164	0.933(0.571-1.524)	0.782
Cesarean section	1.734 (1.280-2.350)	0.001^*^	1.697 (1.153-2.497)	0.007^*^	0.979(0.757-1.267)	0.873
Gender	0.939 (0.741-1.191)	0.605	0.880 (0.662-1.170)	0.378	0.945(0.796-1.121)	0.515
LBW	1.711 (0.833-3.514)	0.144	1.290 (0.562-2.962)	0.548	0.855(0.557-1.313)	0.474
Very LBW	1.550 (0.207-11.588)	0.669	0.150 (0.005-4.532)	0.275	0.282(0.038-2.109)	0.217
Macrosomia	1.201 (0.712-2.024)	0.492	1.042 (0.561-1.935)	0.896	1.000(0.699-1.430)	0.998
SGA	0.863 (0.503-1.483)	0.595	0.745 (0.378-1.467)	0.395	0.918(0.602-1.400)	0.693
Very SGA	1.128 (0.407-3.129)	0.816	0.877 (0.256-3.010)	0.835	0.916(0.451-1.857)	0.807
LGA	1.227 (0.879-1.711)	0.229	1.282 (0.866-1.895)	0.214	1.108(0.887-1.383)	0.366
Very LGA	1.46 (0.808-2.448)	0.228	1.140 (0.595-2.187)	0.693	0.955(0.665-1.372)	0.803
Placenta previa	0.830 (0.454-1.518)	0.545	0.841 (0.397-1.780)	0.651	0.993(0.621-1.587)	0.975
Fetal malformation	1.288 (0.308-5.383)	0.729	1.815 (0.375-8.790)	0.459	1.672(0.769-3.634)	0.194
HDP	2.980 (1.210-7.335)	0.018^*^	1.732 (0.605-4.965)	0.306	0.618(0.367-1.041)	0.071
GDM	0.836 (0.524-1.333)	0.451	0.682 (0.372-1.247)	0.214	0.776(0.518-1.162)	0.218

PTB, preterm birth; LBW, low birth weight; SGA, small for gestational age; LGA, large for gestational age; OR, odds ratio; HDP, hypertensive disorders of pregnancy; GDM, gestational diabetes mellitus.

^1^Adjusted for maternal age at oocyte retrieval, body mass index, cause of infertility, infertility duration, ART procedure types, the level of FSH, number of transferred embryos, stage of embryo development, PGT treatment.

^*^P<0.05.

What’s more, we performed sub-analysis based on maternal age to explore the influence of different FET cycle endometrial protocols on perinatal outcomes in women < 35 years old (**Table 5**) or ≥ 35 years old (**Table S1**). The findings of our study showed that the percentage of cesarean delivery was still significantly higher in HRT group (aOR=1.838; 95%CI, 1.333-2.535) and GnRHa+HRT group (aOR=1.737; 95%CI, 1.157-2.608) than that in NC group for women under 35 years old. There were significantly increased risks of HDP in HRT group than that in NC group (aOR=3.362; 95%CI, 1.059-10.675) among the young subpopulation. However, there were no significant differences between the obstetric and neonatal outcomes and different endometrial preparation protocols for women ≥ 35 years old.

**Table 5:** Crude and adjusted ORs for perinatal outcomes in different endometrial preparation regimens of women under 35 years old.

	NC vs HRT		NC vs GnRHa+HRT		HRT vs GnRHa+HRT	
	Crude OR (95%CI)	Adjusted OR ^1^ (95%CI)	Crude OR (95%CI)	Adjusted OR ^1^ (95%CI)	Crude OR (95%CI)	Adjusted OR^1^ (95%CI)
PTB	1.280 (0.735-2.228)	1.284 (0.736-2.241)	1.536 (0.822-2.868)	1.539 (0.809-2.928)	1.200 (0.857-1.680)	1.206 (0.859-1.694)
Very PTB	2.252 (0.709-7.153)	2.251 (0.707-7.165)	1.794 (0.496-6.494)	1.938 (0.513-7.319)	0.797 (0.425-1.495)	0.801 (0.425-1.508)
Cesarean section	1.826 (1.330-2.506)	1.839 (1.334-2.535) ^*^	1.787 (1.205-2650) ^*^	1.733 (1.154-2.601) ^*^	0.979 (0.748-1.280)	0.949 (0.723-1.246)
Gender	0.867 (0.664-1.131)	0.875 (0.669-1.143)	0.787 (0.574-1.079)	0.772 (0.559-1.068)	0.908 (0.749-1.102)	0.907 (0.747-1.103)
LBW	1.322 (0.612-2.858)	1.313 (0.605-2.847)	1.183 (0.483-2.890)	1.102 (0.441-2.752)	0.895 (0.536-1.493)	0.879 (0.525-1.473)
Very LBW	0.903 (0.118-6.899)	0.940 (0.119-7.413)	0.483 (0.030-7.760)	0.222 (0.10-4.769)	0.535 (0.070-4.077)	0.481 (0.063-3.692)
Macrosomia	1.040 (0.596-1.815)	1.036 (0.591-1.817)	1.003 (0.520-1.938)	0.922 (0.468-1.818)	0.965 (0.647-1.438)	1.007 (0.672-1.508)
SGA	0.973 (0.506-1.873)	0.954 (0.494-1.839)	0.967 (0.445-2.101)	0.878 (0.390-1.978)	0.994 (0.617-1.600)	0.972 (0.601-1.571)
Very SGA	0.869 (0.312-2.422)	0.801 (0.285-2.248)	0.845 (0.245-2.917)	0.660 (0.179-2.427)	0.972 (0.440-2.149)	0.977 (0.439-2.175)
LGA	1.075 (0.751-1.538)	1.065 (0.741-1.530)	1.133 (0.746-1.721)	1.112 (0.722-1.712)	1.054 (0.823-1.351)	1.106 (0.859-1.423)
Very LGA	1.348 (0.725-2.507)	1.357 (0.726-2.536)	1.200 (0.584-2.464)	1.084 (0.518-2.270)	0.890 (0.590-1.343)	0.932 (0.615-1.414)
Placenta previa	0.753 (0.376-1.507)	0.748 (0.372-1.502)	0.801 (0.345-1.858)	0.784 (0.326-1.884)	1.064 (0.614-1.843)	1.050 (0.604-1.827)
Fetal malformation	1.229 (0.295-5.125)	1.219 (0.291-5.105)	0.968 (0.176-5.325)	0.982 (0.171-5.623)	0.788 (0.280-2.218)	0.853 (0.301-2.420)
HDP	3.163 (1.000-10.002)	3.360 (1.059-10.663) ^*^	1.462 (0.392-5.450)	1.650 (0.422-6.455)	0.462 (0.234-0.912) ^*^	0.511 (0.257-1.013)
GDM	1.059 (0.568-1.976)	1.058 (0.565-1.980)	0.967 (0.461-2.030)	1.066 (0.500-2.277)	0.913 (0.580-1.437)	0.936 (0.592-1.479)

PTB, preterm birth; LBW, low birth weight; SGA, small for gestational age; LGA, large for gestational age; OR, odds ratio; HDP, hypertensive disorders of pregnancy; GDM, gestational diabetes mellitus.

^1^Adjusted for body mass index, cause of infertility, infertility duration, ART procedure types, the level of FSH, number of transferred embryos, stage of embryo development, PGT treatment.

^*^P<0.05.

## Discussion

Our study aimed to examine the impact of three most frequently utilized endometrial preparation regimens on the perinatal outcomes in FET cycles. Our findings indicate that HRT cycles, with or without GnRHa pretreatment, were found to be prevalent to a higher incidence of HDP and cesarean section than that of natural cycles in ovulatory women. Sub-analysis of women < 35 years old showed consistent results while no significant differences were detected in terms of maternal and neonatal outcomes among three endometrial preparation protocols for women aged 35 years or older. Besides, compared with HRT cycles and NC cycles, women capable of ovulation didn’t benefit from HRT + GnRHa pretreatment when concerning perinatal outcomes of singletons.

HDP is recognized as the second leading cause of maternal and neonatal morbidity around the world. The prevalence of HDP has doubled from 6.0% to 12.0% between 2000 and 2018 (20). Pregnancy with HDP has a significant impact on newborns, potentially resulting in growth restriction and prematurity (4). Our study’s findings align with several recently published studies examining perinatal outcomes in FET cycles utilizing various endometrial preparation regimens (7, 8, 21–26). A cohort study in Denmark also demonstrated the risks of postpartum hemorrhage and HDP was significantly higher after artificial cycles in comparison to NC (true natural cycles and modified natural cycles) (7). In this regard, after adjusting for confounding variables, our findings confirmed the relationship between HDP and endometrial preparation by hormonal replacement. These results also emphasized the importance of corpus luteum (CL) in the gestational period. In HRT-FET cycles, exogenous estrogen (E2) and progesterone (P) were administered to promote endometrium development, which leads to the suppression of ovary, and thus ovulation doesn’t occur, and there is no CL. CL can generate not only E2 and P, but also vasoactive substances such as vascular endothelial growth factor (VEGF), relaxin (4, 11, 27, 28). These factors play a crucial role in enhancing maternal cardiovascular adaptability during early pregnancy by increasing blood flow. For instance, relaxin, secreted solely by the CL during early pregnancy, has the ability to regulate uterine artery compliance and increase uterine blood flow (27). Compared with NC cycles, HRT cycles are deficient in vasoactive and angiogenic regulatory substances, which may be one of the causes of higher blood pressure during pregnancy. According to a previous study, impaired carotid-femoral pulse wave velocity, indicative of reduced aortic compliance, can serve as a predictive marker for pre-eclampsia. However, the study revealed that pregnant patients who underwent HRT cycles exhibited more blunted carotid-femoral pulse wave velocities compared to those undergoing NC cycles (25).

Our study failed to demonstrate the difference in newborn birthweight across different FET regimens. Notably, significant differences were not detected in terms of LBW, macrosomia, SGA, very SGA, LGA and very LGA among various endometrial preparation protocols. Ginström Ernstad et al. reported there were no significant differences in the risks of SGA and LGA in natural, stimulated and artificial cycles (24), which was in accordance with our results to some extent. Another RCT study conducted in Netherlands showed women undergoing NC or HRT cycles didn’t affect the birthweight of newborns significantly (29). However, there are few available studies suggesting a higher incidence of LGA and macrosomia in HRT cycles (7, 9, 21). In HRT cycles, exogenous estrogen was applied to develop endometrium. Previous studies have demonstrated that the administration of supra-physiological levels of estrogen could potentially yield unfavorable consequences on perinatal outcomes (30, 31). In contrast, the duration of estrogen didn’t affect newborn birthweight in HRT-FET cycles (32). Gluckman PD et al. declared that newborns with abnormal birthweight were more likely to become obese and have adverse chronic diseases in the future (33). The results of our study aren’t able to approve the hypothesis that deficiency of hormonal stimulation leads to better neonatal outcomes concerning newborn birthweight. We speculated the potential explanations may due to the distinctive patients’ traits, as we focused on ovulatory women whose BMI < 28 kg/m2 and age ≤ 42 years old in order to be homogenous while other studies include both ovulatory and anovulatory patients.

It is noteworthy that the global incidence of cesarean section has significantly risen in recent years. The overall percentage of cesarean section in China was 54.9% (34). In the present study, there is an increased caesarean section rate in HRT cycles and HRT + GnRHa cycles than in NC cycles, which is consistent with prior research (35, 36). We assumed this result may be attributed to the higher incidence of obstetric complications such as HDP, which may increase the likelihood of caesarean section when delivering.

Another interesting result of our study is that higher risks of HDP and cesarean section in HRT cycles over NC cycles for young ovulatory women under the age of 35. Conversely, this trend was not observed in women aged 35 years or older. Our finding provides supportive evidence for selecting an appropriate endometrial preparation strategy for ovulatory women of varying age. For younger patients in FET, NC cycles may be a more suitable option to mitigate potential risks. As far as we know, previously published studies focused on the adverse perinatal and neonatal outcomes of artificial cycles for women undergoing FET regardless of their age (7, 21). Furthermore, the findings of our study assumed HRT cycles and HRT with GnRHa pretreatment may have adverse effects on young women, highlighting the importance of considering female age when selecting endometrial protocols. It can also provide evidences for clinical physicians in determining the optimal endometrial regimens based on patients’ characteristics.

In our study, we included NC cycles and HRT cycles to clarify the association between perinatal outcomes and endometrial preparation regimens, and further categorized HRT cycles into two groups:GnRHa+HRT and HRT alone, while previously published studies failed to divide HRT cycles into subgroups based on with GnRHa pretreatment or not (37–39). GnRHa pretreatment were mainly used to women with recurrent implantation failure (RIF), endometriosis (EMT) and adenomyosis. The administration of GnRHa can deregulate the pituitary gland and lead to hypogonadotropic-hypogonadal state, which can result in prolonged amenorrhea and reduced level of estradiol (40). Previous studies have attempted to investigate the potential benefits of GnRHa pretreatment in HRT cycles, but definitive conclusions have yet to be drawn. Only one RCT has reported that GnRHa pretreatment achieved better pregnancy outcomes with increased clinical pregnancy rates and live birth rates (41), while other findings held the idea that patients weren’t able to benefit from GnRHa pretreatment in HRT cycles (14, 42, 43). A multicenter study from China suggested that GnRHa+HRT had higher preeclampsia risk than HRT alone, possibly due to its stronger suppression of ovulation than in HRT cycles (44). However, studies mentioned above have primarily focused on pregnancy outcomes and specific population. Furthermore, compared with NC cycles and HRT cycles, whether GnRHa pretreatment in HRT cycles can improve perinatal outcomes for ovulatory women is equivocal. One RCT compared HRT with and without GnRHa administration previously for PCOS women, and drew the conclusion that GnRHa pretreatment in HRT not only added costs but also increased absolute cycles cancellation rates, however, provided no pregnancy-related benefits (13). Besides, several mechanism studies that have demonstrated GnRHa improved endometrium receptivity were conducted on animals (45, 46). At present study, we found GnRHa pretreatment in HRT cycles had no superiority over HRT cycles in perinatal outcomes.

The primary strength for this study lies in the large sample size of singleton deliveries and the integrity of baseline from a single-center. The center’s procedures for ovarian stimulation, IVF/ICSI regimens and conditions of laboratory enhance the reliability of our study. In addition, we conducted univariable and multivariable regression models to minimize the impact brought by bias. Moreover, we established strict inclusion and exclusion criteria to ensure homogeneity among the enrolled population, limiting it to individuals capable of ovulation and with regular menstruation. To mitigate the influence of maternal BMI on perinatal outcomes, restrictions were implemented. This was due to the higher likelihood of overweight women developing LGA and PTB, as previously reported (47). However, as the study was retrospective, there was no doubt that some inherent biases existed inevitably. For instance, the clinicians chose endometrial preparation regimens based on individual patient characteristics and personal preferences, leading to the imbalanced distribution of included patients across the three groups. Therefore, prospective studies are warranted to ascertain to ascertain the effect of endometrial preparation protocols in the near future.

## Conclusion

In conclusion, our findings enhanced previous studies addressing that HRT cycles with or without GnRHa pretreatment are related to an increased risks of HDP and cesarean section, compared with NC cycles, especially for ovulatory women under 35 years old. There were no differences in infants’ birthweight including LBW, macrosomia, SGA, very SGA, LGA, very LGA among different FET endometrial preparation protocols. Additionally, our study also demonstrated that HRT with GnRHa pretreatment showed no advantages over HRT cycles on perinatal outcomes of ovulatory women.

## Data availability statement

The raw data supporting the conclusions of this article will be made available by the authors, without undue reservation.

## Ethics statement

The studies involving humans were approved by Review Board of Tongji Hospital, Tongji Medical College, Huazhong University of Science and Technology. The studies were conducted in accordance with the local legislation and institutional requirements. Written informed consent for participation was not required from the participants or the participants’ legal guardians/next of kin in accordance with the national legislation and institutional requirements.

## Author contributions

KQ and YG designed the study. XH, ZL, YZ, and JL collected the data. XH performed the analysis of the data and wrote the manuscript. All authors contributed to the article and approved the submitted version.
