# Supplementary material for: Young women were associated with higher risk of hypertensive disorders of pregnancy and cesarean section from hormone replaced cycles in frozen-thawed embryo transfer: a retrospective study of 5316 singleton deliveries
Source: Front Endocrinol (Lausanne). 2023 Sep 5;14:1238887. doi: 10.3389/fendo.2023.1238887 (PMC10523772; doi:10.3389/fendo.2023.1238887)
Supplement: Supplementary file 1 [file DataSheet_1.docx]

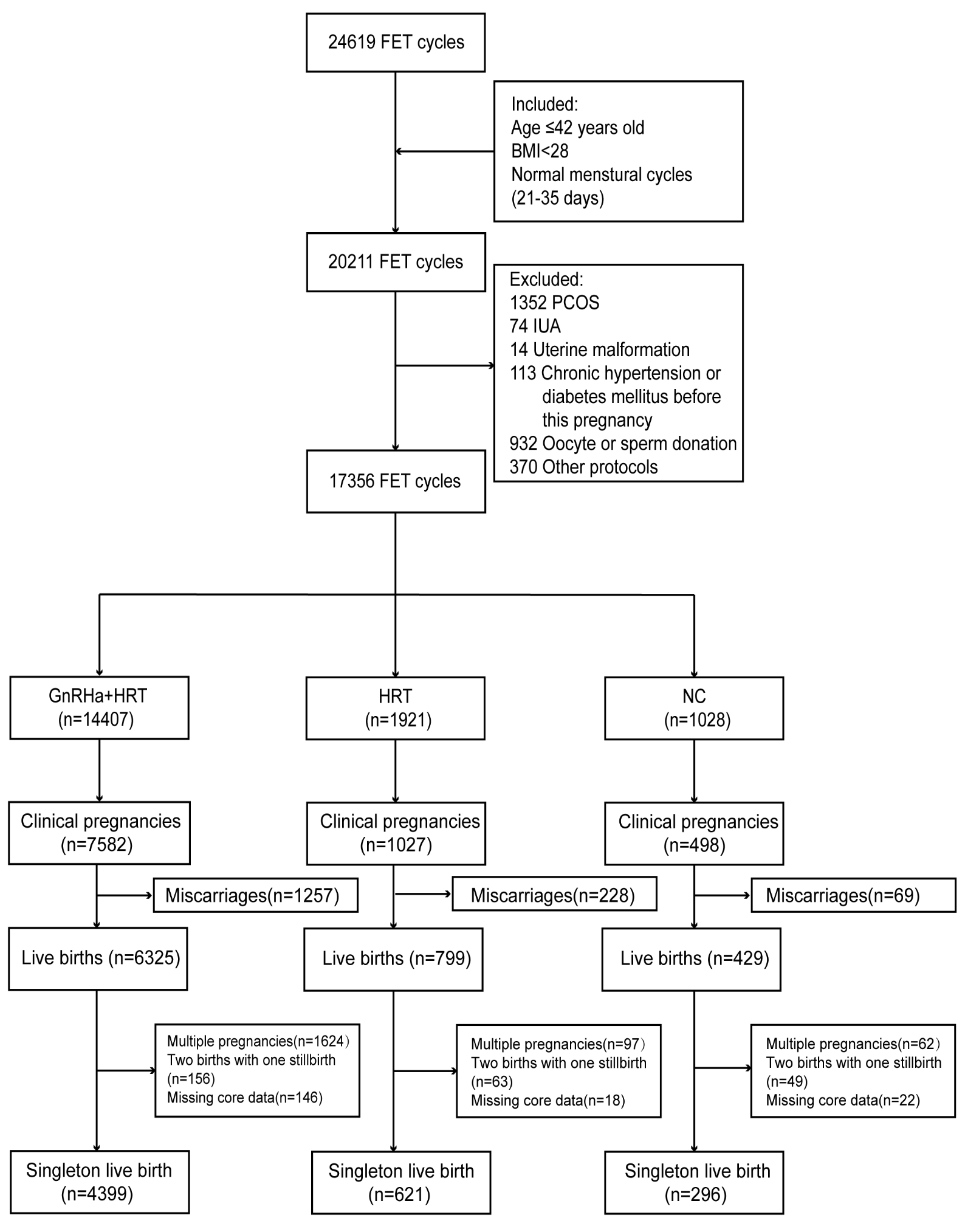
Supplementary figure 1: Flowchart of patients

Supplementary table 1: Crude and adjusted ORs for perinatal outcomes in different endometrial preparation regimens of women ≥35 years old

|  | NC vs HRT | | NC vs GnRHa+HRT | | HRT vs GnRHa+HRT | |
| --- | --- | --- | --- | --- | --- | --- |
|  | Crude OR (95%CI) | Adjusted OR (95%CI) | Crude OR (95%CI) | Adjusted OR (95%CI) | Crude OR (95%CI) | Adjusted OR (95%CI) |
| PTB | 1.167 (0.490-2.782) | 1.248 (0.518-3.003) | 0.976 (0.354-2.693) | 1.093 (0.363-3.296) | 0.836 (0.454-1.541) | 0.950 (0.486-1.683) |
| Very PTB | 1.489 (0.351-6.319) | 1.583 (0.368-6.809) | 1.853 (0.382-8.978) | 2.489 (0.451-13.744) | 1.245 (0.567-2.731) | 1.324 (0.597-2.939) |
| Cesarean section | 0.930 (0.326-2.654) | 0.949 (0.330-2.733) | 1.283 (0.362-4.547) | 1.786 (0430-7.419) | 1.380 (0.615-3.095) | 1.364 (0.601-3.098) |
| Gender | 1.290 (0.768-2.166) | 1.198 (0.708-2.026) | 1.354 (0.745-2.461) | 1.451 (0.769-2.736) | 1.050 (0.736-1.498) | 1.047 (0.728-1.505) |
| LBW | 4.244 (0.577-31.220) | 4.368 (0.589-32.376) | 3.765 (0.461-30.743) | 2.753 (0.292-26.008) | 0.887 (0.412-1.910) | 0.813 (0.372-1.778) |
| Very LBW | / | / | / | / | / | / |
| Macrosomia | 2.177 (0.518-9.141) | 2.501 (0.586-10.679) | 1.853 (0.382-8.978) | 2.293 (0.429-12.250) | 0.851 (0.396-1.830) | 0.924 (0.424-2.015) |
| SGA | 0.664 (0.254-1.739) | 0.629 (0.238-1.665) | 0.522 (0.153-1.776) | 0.316 (0.070-1.430) | 0.786 (0.328-1.881) | 0.692 (0.283-1.692) |
| Very SGA | / | / | / | / | 0.811 (0.182-3.606) | 0.722 (0.157-3.310) |
| LGA | 2.166 (0.918-5.110) | 2.299 (0.959-5.508) | 2.167 (0.845-5.553) | 3.110 (1.098-8.8130 | 1.000 (0.631-1.585) | 1.064 (0.663-1.707) |
| Very LGA | 1.486 (0.452-4.887) | 1.547 (0.463-5.166) | 1.378 (0.360-5.266) | 1.812 (0.409-8.032) | 0.927 (0.448-1.921) | 0.974 (0.463-2.050) |
| Placenta previa | 1.004 (0.301-3.346) | 0.981 (0.290-3.311) | 0.899 (0.218-3.710) | 0.911 (0.200-4.159) | 0.895 (0.371-2.157) | 0.993 (0.408-2.418) |
| Fetal malformation | / | / | / | / | / | / |
| HDP | 2.045 (0.486-8.603) | 2.544 (0.595-10.876) | 1.609 (0.325-7.968) | 1.540 (0.252-9.397) | 0.787 (0.349-1.772) | 0.808 (0.354-1.846) |
| GDM | 0.513 (0.251-1.051) | 0.551 (0.265-1.148) | 0.239 (0.083-0.690) | 0.230 (0.069-0.770) | 0.466 (0.198-1.093) | 0.520 (0.219-1.232) |

PTB = preterm birth; LBW = low birth weight; SGA = small for gestational age; LGA = large for gestational age; OR = odds ratio; HDP = hypertensive disorders of pregnancy; GDM = gestational diabetes mellitus

^*^P<0.05
